# Urinary *β*2-Microglobulin Is a Good Indicator of Proximal Tubule Injury: A Correlative Study with Renal Biopsies

**DOI:** 10.1155/2014/492838

**Published:** 2014-11-20

**Authors:** Xu Zeng, Deloar Hossain, David G. Bostwick, Guillermo A. Herrera, Ping L. Zhang

**Affiliations:** ^1^Department of Pathology and Laboratory Medicine, Temple University Hospital, 3401 North Broad Street, Room A2-F326, 2PAP, Philadelphia, PA 19140, USA; ^2^Bostwick Laboratories, Orlando, FL 32809, USA; ^3^Department of Pathology, Louisiana State University Health Sciences Center, Shreveport, LA 71103, USA; ^4^Department of Pathology, William Beaumont Hospital, Royal Oak, MI 48073, USA

## Abstract

*Objective*. After filtration through glomeruli, *β*2-microglobulin is reabsorbed in proximal tubules. Increased urinary *β*2-microglobulin indicates proximal tubule injury and measurement of *β*2-microglobulin in urine is useful to determine the source of renal injury. Kidney injury molecule-1 (KIM-1) has been characterized as a selective proximal tubule injury marker. This study was designed to evaluate the correlation of urinary *β*2-microglobulin concentration and KIM-1 expression as evidence of proximal tubule injury. *Methods*. Between 2009 and 2012, 46 patients with urine *β*2-microglobulin (RenalVysion) had follow-up kidney biopsy. Diagnoses included glomerular and tubule-interstitial disease. Immunohistochemical staining for KIM-1 was performed and the intensity was graded from 0 to 3+. Linear regression analysis was applied to correlate the values of urinary *β*2-microglobulin and KIM-1 staining scores. *P* < 0.05 was considered statistically significant. *Results*. Thirty patients had elevated urinary *β*2-microglobulin. KIM-1 staining was positive in 35 kidney biopsies. There was a significant correlation between urinary *β*2-microglobulin and KIM-1 staining (*P* < 0.05). Sensitivity was 86.6%, specificity was 43.7%, positive predictive value was 74.2%, and negative predictive value was 63.6%. *Conclusion*. Increased urinary *β*2-microglobulin is significantly correlated with KIM-1 staining in injured proximal tubules. Measurement of urine *β*2-microglobulin is a sensitive assay for proximal tubule injury.

## 1. Introduction

Acute kidney injury is a common clinical syndrome that is characterized by a rapid decline in kidney function, often triggered by glomerular disease and/or tubulointerstitial disease and associated with high morbidity and mortality [1–4]. In addition to serum creatinine, *β*2-microglobulin is a biomarker that can be used to determine underlying causes of acute kidney injury [5]. Serum *β*2-microglobulin derives from cellular membrane turnover [6], since *β*2-microglobulin forms the invariant light chain portion of major histocompatibility complex (MHC) class I in membranes of all cells [7–10]. As a single-chain small polypeptide (MW = 11.8 kDA), *β*2-microglobulin is filtered almost completely through the glomeruli of the healthy kidney [11, 12] and then reabsorbed by the renal proximal tubules. Only a small amount of *β*2-microglobulin can be detected in the urine under normal physiological conditions. Therefore, levels of serum and urinary *β*2-microglobulin reflect the functions of glomeruli and proximal tubules [13]. In patients with acute kidney injury, an increase in serum creatinine, together with an increase in urinary *β*2-microglobulin, strongly suggests proximal tubule injury. On the other hand, an increase in serum creatinine with elevated serum *β*2-microglobulin is seen in patients with decrease in glomerular filtration rate [14]. Elevated urinary *β*2-microglobulin was associated with tubular injury caused by viral infection, ischemia, and toxicity from medications or heavy metals [15, 16].

Measurement of urinary *β*2-microglobulin is convenient and has been applied in routine clinical practice (RenalVysion Bostwick Laboratories, Uniondale, NY) to evaluate the tubular function [17]. Using immunohistochemical staining for CD133 in kidney biopsies, we demonstrated that urinary *β*2-microglobulin is a sensitive indicator for tubular injury [5]. However, a direct correlation between urinary *β*2-microglobulin and proximal tubule injury has not yet been fully established due to lack of a specific proximal tubule injury marker.

Recently, kidney injury molecule-1 (KIM-1) was shown to be a biomarker for proximal tubule injury [18–20]. KIM-1 is a type 1 cell membrane glycoprotein comprised of extracellular, transmembrane and intracellular domains [21, 22]. KIM-1 is undetectable in the healthy kidney [19]. With different forms of kidney injury, mRNA and protein levels of KIM-1 are upregulated and expressed [20, 23, 24] in injured tubules [23, 25–27]. KIM-1 binds to surface-specific epitopes on the apoptotic bodies and cellular debris derived from injured tubular cells, facilitating macrophages to engulf dying cells [19]. In addition, the extracellular domain of KIM-1 can be cleaved by metalloproteinases into a soluble shedded part and short membrane bound fragments that are released into urine. Therefore, the presence of KIM-1 in tubular epithelium and/or urine indicates tubule injury. Moreover, the expression of KIM-1 in injured tubule cells is limited to the proximal tubular cells [25]. Using immunohistochemistry, KIM-1 staining in kidney tissue has been shown to accurately detect proximal tubular injury associated with ischemic and toxic type of tubular injury, allograft rejection in renal transplantation, and acute pyelonephritis [20, 26, 28].

The present study was designed to examine the correlation between increase in urinary *β*2-microglobulin and proximal tubule injury assessed by KIM-1 staining in kidney biopsies.

## 2. Materials and Methods

Between January 2009 and December 2012, a total of 5494 patients had urinary *β*2-microglobulin measurements by RenalVysion analysis (Bostwick Laboratories). Among these, fifty-six patients had follow-up renal biopsies. Nine renal biopsies either contained less than 10 glomeruli per histological section or had insufficient tissue to perform immunohistochemical staining and were excluded from the present study. One biopsy showed high background staining and was also excluded. The remaining forty-six biopsies met selection criteria and were included in the present study.

Urine collection followed the instruction of RenalVysion. Briefly, 10 mL fresh urine was collected from each patient and urine pH was adjusted to pH 6–8 with 1 M NaOH. The specimen was delivered to lab by overnight shipping and the level of *β*2-microglobulin was measured next day. The majority of immunohistochemical staining was performed in archived unstained slides prepared at the time of renal biopsy. In those biopsies in which unstained slides were not available, additional new slides were prepared from archival tissue from formalin-fixed paraffin-embedded blocks. All sections were cut at 3 *μ*m. Each slide contained at least 2 sections.

Immunohistochemical staining for KIM-1 was conducted on paraffin slides using Dako autostainer (Dakocytomation, Carpinteria, CA). To remove paraffin, sections were heated at 60°C for 60 min, placed into xylene baths for 3 min, 3 times, followed by 100% alcohol, for 3 min, 3 times, and then washed by running water for 30 s. Antigen retrieval was carried out using a Tris-EDTA buffer at pH 8.0 for 20 min at 99°C and cooled down for 20 min at room temperature, raised by tap water. Slides were placed into H_2_O_2_ for 15 min and then placed in Tris buffer (pH 7.6). Finally, slides were placed into Dako autostainer programmed by Thermo Scientific UltraVision LP Detection System (Kalamazoo, MI). Staining procedures included 5 min Ultra V block, 30 min incubation with primary KIM-1 monoclonal antibody (AKG7 clone at 1 : 10 dilution, kindly provided by Dr. Joseph V. Bonventre from renal division Brigham and Women's Hospital, Boston), 8 min primary antibody enhancer, 10 min HRP polymer (equivalent to secondary antibody), and 5 min of chromogen DAB to achieve brown staining.

Granular staining in the cytoplasm and membrane of proximal tubular epithelium was considered positive. Staining intensity of KIM-1 was scored from 0 to 3+ as follows: 0, absence of staining; 0.5, focal weak fine granular staining; 1+, weak fine granular staining cytoplasm and/or membrane in entire tubular luminal surface; 2+, moderate granular staining; and 3+, strong coarse granular staining.

Patient charts were retrospectively reviewed. Demographic information, including age and gender, was recorded for each patient. Parameters related to tubular injury including serum creatinine, granular and cellular casts, and urine cytological findings were also recorded. Staining with KIM-1 was evaluated by two pathologists who were blinded to the clinical data, final pathological diagnosis and patient outcome.

Linear regression analysis was used to correlate values of urinary *β*2-microglobulin and KIM-1 staining scores. *P* value < 0.05 was considered statistically significant. Odds ratio analysis was applied to express the association of urinary *β*2-microglobulin level and intensity of KIM-1 staining. Data were also stratified according to urinary *β*2-microglobulin level as normal (<300 *μ*g/L) versus increased to KIM-1 staining (positive or negative) and evaluated by 2 × 2 contingency table (Table 2). The sensitivity, specificity, predictive value for positive result, and predictive value for negative result were calculated as follows:

 sensitivity = true positive/(true positive + false negative);
 specificity = true negative/(true negative + false positive);
 predictive value for positive result = true positive/(true positive + false positive);
 predictive value for negative result = true negative/(true negative + false negative);

The age of the patients was expressed as mean ± SD.

## 3. Results

**Figure 1:**
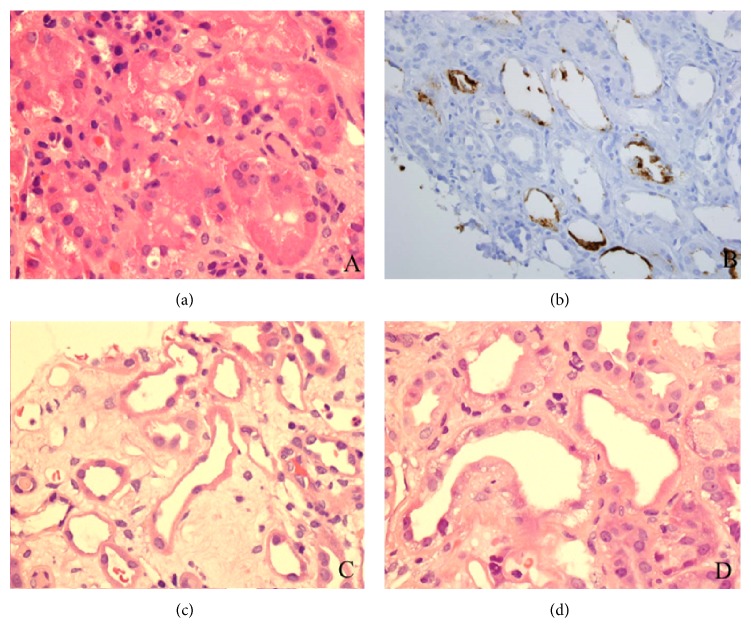
(a) Normal proximal tubules with back to back proximal tubules and abundant cytoplasm (H&E 40 × 10). (b) In addition to flattening and simplification of tubular epithelium, injured proximal tubules show granular cytoplasmic and membrane staining for KIM-1 (20 × 10). (c) Ischemia type of tubular injury shows flattening and simplification of tubular epithelium (H&E 40 × 10). (d) In addition to flattened tubular epithelium, toxic type of tubular injury reveals vacuoles in cytoplasm (H&E 40 × 10).

Among 5494 patients with urinary *β*2-microglobulin measurement, renal biopsy was obtained out in 56 patients (56/5494, 1.0%). Among these, 46 biopsies met the selection criteria and were included in present study. Thirty patients had increases in urinary *β*2-microglobulin; it was in normal range in the remaining 16 patients. The demographic details for 35 patients (M : F = 22 : 13) with positive KIM-1 staining in proximal tubules are shown in Table 1. The age of patients at the time of biopsy was 54.9 ± 10.7 years ranging from 16.0 to 87.0 years. The majority of renal biopsies were performed in native kidneys. Two biopsies were from renal transplant patients. A majority of renal biopsies showed glomerular disease. Nine patients had combined glomerular and tubulointerstitial involvement. Glomerular diseases included membranous nephropathy, antineutrophil cytoplasmic antibody- (ANCA-) associated glomerulonephritis, diabetic nephropathy, focal and segmental glomerulosclerosis, IgA nephropathy, and lupus nephritis. Four patients had acute interstitial nephritis. In addition to glomerular disease, five patients had tubular injury (Figures 1(c) and 1(d)), two of which were toxic type of injury with cytoplasmic vacuoles (Figure 1(d)). The remaining three biopsies showed ischemic tubular injury.

Of the 26 patients with elevated urinary *β*2-microglobulin and positive KIM-1 staining in proximal tubules, 18 patients had increased serum creatinine, ranging from 1.50 mg/dL to 6.00 mg/dL (Table 1). Since the elevated urinary *β*2-microglobulin is associated with proximal tubular injury [14], in these 18 patients it was considered that the proximal tubular injury is the primary cause of increase in serum creatinine. Nine patients with normal urinary *β*2-microglobulin had positive KIM-1 staining in proximal tubules, 6 of which had increase in serum creatinine, ranging from 1.10 mg/dL to 3.20 mg/dL. Granular casts were presented in 2 patients, who also had increase in both urinary *β*2-microglobulin and serum creatinine. An increase in tubular epithelial cells was seen in 19/35 (54%) patients. Eleven of 26 patients also had microscopic hematuria.

**Figure 2:**
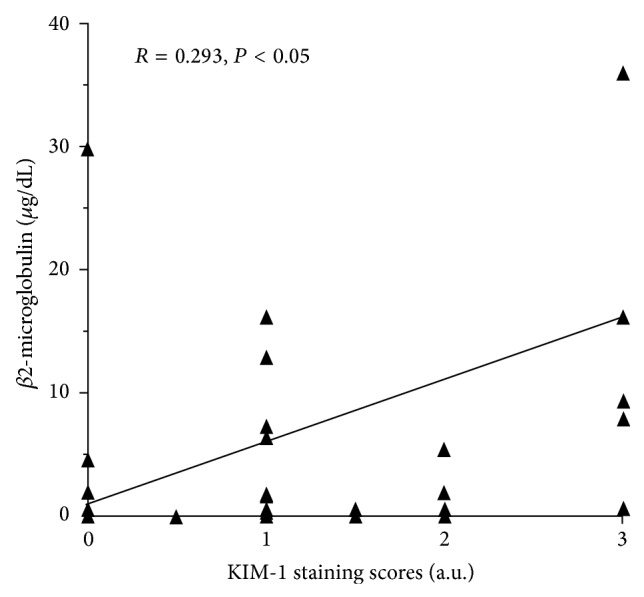
Linear regression analysis shows a significant correlation between levels of urinary *β*2-microglobulin and KIM-1 staining scores (*R* value = 0.293, *P* < 0.05). Odds ratio was 7.229. Confidence interval at 95% range was 2.868 to 12.01.

Thirty-five of 46 biopsies showed positive KIM-1 staining with a granular pattern in the cytoplasm and/or apical membrane of tubular epithelium, ranging from finely granular to coarsely granular (Figure 1(b)). Positive staining for KIM-1 was found in proximal tubules only. No staining in distal tubules, glomeruli, or vessels was seen. The corresponding H&E slides of kidney biopsy showed flattening and simplification of tubular epithelium, ranging from focal to diffuse, overall focal tubular injury with varying degree, and mild tubular injury in the majority of the kidney biopsies. Staining intensity ranged from 0.5+ to 3+ with 1+ staining in 18 patients (18/35, 51.4%). The relation between value of urinary *β*2-microglobulin and KIM-1 staining score is shown in Figure 2. There was a significant correlation between levels of urinary *β*2-microglobulin and KIM-1 staining (*R* value = 0.293, *P* < 0.05) (Figure 2). Odds ratio was 7.229, implying that levels of urinary *β*2-microglobulin are strongly associated with intensity of KIM-1 staining. Confidence interval at the 95% range was 2.868 to 12.01.

Among 35 biopsies with KIM-1 positive staining, 26 biopsies had increase in urinary *β*2-microglobulin. The remaining 9 biopsies had normal urinary *β*2-microglobulin (Table 2). Using positive KIM-1 staining as the end point, sensitivity was 86.6%, specificity was 43.7%, positive predictive value was 74.2%, and negative predictive value was 63.6%.

## 4. Discussion

We found that increased urinary *β*2-microglobulin was significantly correlated with KIM-1 staining in proximal tubules, indicating that urinary *β*2-microglobulin is a sensitive (sensitivity 86.6%) assay for detecting tubular injury. Further, because KIM-1 is a selective proximal tubule injury marker, the present study also demonstrated that measurement of urinary *β*2-microglobulin was a reliable assay to detect proximal tubule injury. Therefore, in patients with acute kidney injury with increase in serum creatinine, measurement of urinary *β*2-microglobulin is also helpful to identify the underlying tubular dysfunction, that is, proximal tubule injury.

Acute tubular injury is a common clinical diagnosis. In a patient with classic clinical presentations such as hypotension together with a rapid rising of serum creatinine, the diagnosis of severe tubular injury (acute tubular necrosis) can be made and does not require kidney biopsy to initiate treatment. However, the diagnosis of glomerular disease accompanied by tubular injury is not easy to establish, as both may contribute to elevated serum creatinine. As shown in Table 1, the majority of kidney biopsies showed glomerular disease. Tubular injury was the secondary diagnosis in 5/35 patients but other evidence of tubular injury such as tubular casts and an increase in tubular cells by urine analysis was found in 22/35 patients. Moreover, 2 of 4 patients with secondary diagnosis of acute interstitial nephritis showed tubulitis, a feature associated with tubular injury. These findings indicate that glomerular diseases associated with varying degree of tubular injury could be missed in renal biopsies due to sampling. In a prior study, using CD133 staining in injured tubular epithelium, it was demonstrated that urinary *β*2-microglobulin was a sensitive indicator for tubular injury [5]. As a progenitor marker, CD133 positive cells were scattered along the tubular epithelium. Thus, confluent CD133 staining is required to diagnose tubular injury. In addition, CD133 positive cells are present not only in proximal tubular cells but also in distal tubular cells and/or atrophic tubules. Therefore, a careful morphological correlation is needed to exclude false positive staining. By comparison, KIM-1 is a selective proximal tubule injury marker that only presents in patients with proximal tubule injury [18–20]. No staining for KIM-1 was seen in healthy tubular epithelium. No distal tubular cell or atrophic tubular cell staining was identified. Therefore, KIM-1 is a more specific marker than CD133 for detecting proximal tubular injury. In the present study, KIM-1 was able to detect patients with mild, focal tubular injury evidenced by urine analysis as an increase in number of tubular cells; some patients presented with normal levels of serum creatinine (Table 1). This was also supported with the finding in the present study that more than half of KIM-1 staining score was weakly positive (1+). These findings highlight that KIM-1 is a sensitive marker for proximal tubular injury. A good correlation of urinary *β*2-microglobulin and KIM-1 staining (*R* value of 0.293) indicates that measurement of urinary *β*2-microglobulin is a reliable assay for proximal tubule injury. Similar to CD133 staining, KIM-1 was also able to identify two major types of tubular injury: ischemic and toxic type (Figures 1(c) and 1(d)). As shown in Table 2, tubular injury was an additional diagnosis included in 5 of 26 biopsies (25%) (Table 1), and all showed positive KIM-1 staining in proximal tubules.

Using KIM-1 staining as a proximal tubule injury marker in the present study, specificity was lower than the prior study that used CD133 staining [5]. Nine patients, rather than six patients with normal urinary *β*2-microglobulin, presented with positive KIM-1 staining, which were considered false positive. With low specificity, clinical interpretation of elevated urinary *β*2-microglobulin should be closely correlated with clinical history, (e.g., history of hypotension, recent history of medications use, etc.) and clinical presentation, including value of serum creatinine, as well as urine cytological findings. The diagnosis of tubular injury with RenalVysion was made not only based on the elevation of urinary *β*2-microglobulin and serum creatinine but also combined with morphological findings of tubular casts and the number of sloughed tubular epithelial cells in urine analysis.

RenalVysion is a urine-based analysis that combines urine chemistry and cytology to detect glomerular disease and tubular injury. Normal range of urinary *β*2-microglobulin was set between 230 *μ*g/L and 300 *μ*g/L [29]. Elevation of urinary *β*2-microglobulin over 300 *μ*g/L was considered to be abnormal by RenalVysion. Therefore, nine patients with urinary *β*2-microglobulin <300 *μ*g/L and positive KIM-1 staining in biopsies were considered to be false positive. However, the actual values of urinary *β*2-microglobulin in 2 patients were marginally high in 280 *μ*g/L and 290 *μ*g/L. The remaining 7 patients were found to have levels less than 210 *μ*g/L, which is the measurement threshold of the assay. Because the confidence interval at the 95% range in lower end was 2.868 in present study, the normal range of urinary *β*2-microglobulin applied in RenalVysion should be reconsidered to be 2.868 *μ*g/L. If so, the patient with urinary *β*2-microglobulin of 290 *μ*g/L should be considered as abnormal and belongs to true positive for KIM-1 staining. Therefore, corrected sensitivity is 87.1%, specificity is 46.6%, positive predictive value is 77.1%, and negative predictive value is 63.6%.

Our study has multiple limitations. Only a small number of patients had follow-up renal biopsies after RenalVysion (56/5494, 1.0%), raising concern for selection and ascertainment biases. As shown in Table 1, these patients had glomerular disease and/or tubular-interstitial disease. No severe tubular injury as the single diagnosis was seen. The reason for this might be that renal biopsy is usually avoided in patients with a clear clinical diagnosis of severe tubular injury. A larger study cohort involving patients with severe tubular injury is needed to better correlate renal histopathology and immunohistochemistry. Owing to limited sampling by biopsies, a focal tubular lesion might be missed in kidney biopsies chosen in present study. The retrospective nature of the study represents an additional limitation.

In summary, measurement of urinary *β*2-microglobulin is a sensitive assay to screen patients with proximal tubule injury. Our data indicate a correlation of urinary *β*2-microglobulin with results of immunohistochemical staining of KIM-1 in renal biopsy and consequently with proximal tubular injury.

## Figures and Tables

**Table 1:** Clinical indices, diagnosis, concentration of *β*2-microglobulin, serum creatinine, and urine analysis.

Age (year)	Gender	Diagnosis	*β*2-microglobulin (*µ*g/L)	KIM score	Serum creatinine (mg/dL)	Urine analysis
53	F	IgA nephropathy	<0.21	0.5	0.9	Hematuria
72	M	Membranous nephropathy	<0.21	1.5	2.4	↑ Tubular cells, hematuria
59	F	Diabetic nephropathy	<0.21	1.0	1.51	↑ Tubular cells
51	F	Focal, segmental glomerulosclerosis	<0.21	1.0	1.1	Hematuria
46	M	Diabetic nephropathy, IgA nephropathy	<0.21	2.0	2.5	↑ Tubular cells, hematuria
37	M	Lupus nephritis	<0.21	1.5	3.2	↑ Tubular cells, hematuria
72	F	Acute interstitial nephritis, IgA nephropathy	<0.21	2.0	2.3	Hematuria
69	M	Membranous nephropathy	0.28	1.0	0.99	↑ Tubular cells
87	M	IgA nephropathy, acute interstitial nephritis	0.29	2.0	3.2	↑ Tubular cells
50	M	Lupus nephritis, tubular injury (toxic)	0.31	3.0	0.64	
83	M	Diabetic nephropathy, tubular injury	0.33	1.0	1.00	
53	F	Focal, segmental glomerulosclerosis	0.47	1.0	2.40	↑ Tubular cells
60	M	IgA nephropathy, tubular injury	0.49	2.0	5.00	↑ Tubular cells, hematuria
29	M	Renal transplantation, tubular injury	0.53	2.0	4.50	↑ Tubular cells, hematuria
35	M	IgA nephropathy	0.54	2.0	2.90	↑ Tubular cells, hematuria
49	M	Lupus nephritis	0.63	3.0	1.03	↑ Tubular cells
68	M	Focal, segmental glomerulosclerosis	0.69	1.0	1.15	Hematuria
44	F	Focal, segmental glomerulosclerosis	1.15	1.0	1.10	↑ Tubular cells, hematuria
16	F	Focal, segmental glomerulosclerosis	1.60	3.0	2.00	↑ Tubular cells, hematuria
52	F	Granuloma	1.89	2.0	0.70	↑ Tubular cells
50	M	IgA nephropathy	1.89	2.0	0.83	
20	M	Normal	4.52	1.0	3.26	↑ Tubular cells
47	M	Focal, segmental glomerulosclerosis	5.36	2.0	1.18	↑ Tubular cells, hematuria
47	M	Renal transplantation	5.44	2.0	1.19	Hematuria
74	M	Focal, segmental glomerulosclerosis	6.23	3.0	1.50	Granular casts
63	F	Focal, segmental glomerulosclerosis	7.19	1.0	4.50	↑ Tubular cells, hematuria
38	M	Diabetic nephropathy	7.87	2.0	3.00	Granular casts
77	F	ANCA-associated glomerulonephritis	9.23	3.0	2.90	
73	M	ANCA-associated glomerulonephritis	12.9	1.5	2.30	
63	M	Diabetic nephropathy, tubular injury (toxic)	16.1	1.0	6.00	↑ Tubular cells
42	M	Focal, segmental glomerulosclerosis	29.8	1.0	1.70	Neutrophils
62	F	Focal, segmental glomerulosclerosis	35.9	3.0	7.10	↑ Tubular cells
68	F	Focal, segmental glomerulosclerosis	46.3	2.0	2.50	↑ Tubular cells
53	F	Acute interstitial nephritis	47.2	1.0	5.60	↑ Tubular cells
60	M	Acute interstitial nephritis	79.8	2.0	4.50	↑ Tubular cells

**Table 2:** Correlation of urinary *β*2-microglobulin and KIM-1 staining.

	KIM-1 (+)	KIM-1 (−)
*β*2-microglobulin increase (30)	26	4
*β*2-microglobulin normal (16)	9	7
